# The smog of exercise: the causal effect of air pollution on fitness behaviors

**DOI:** 10.3389/fpubh.2025.1692650

**Published:** 2025-10-22

**Authors:** Wen Cao, Tianyu Gao, Chen Ao, Huijun Xiong, Na Li

**Affiliations:** ^1^Non-Performing Asset Management Department, Jiangxi Rural Commercial United Bank Co., Ltd., Nanchang, China; ^2^School of Accounting, Jiangxi University of Finance and Economics, Nanchang, China; ^3^School of Economics, Jiangxi University of Finance and Economics, Nanchang, China; ^4^School of Public Finance and Public Administration, Shanghai Lixin University of Accounting and Finance, Shanghai, China; ^5^School of Economics and Management, Jiangxi Agricultural University, Nanchang, China

**Keywords:** air pollution, fitness behaviors, temperature inversion, two-stage least squares, causal effect

## Abstract

With the rapid development of industrialization and urbanization in China, air pollution has become an increasingly serious problem, and people are increasingly concerned about its impact on personal physical and mental health. From the perspective of health behavior, this study investigates the participation of residents in sports activities by examining fitness behavior and using temperature inversion as an instrumental variable for air pollution. Based on the China Family Panel Studies dataset from 2010 to 2018, this paper empirically examines the causal relationship between air pollution and residents’ participation in fitness activities using two-stage least squares. The research results reveal a statistically significant negative correlation: as air pollution intensifies, the likelihood of individuals participating in fitness activities decreases. Additionally, the research results highlight the heterogeneous effects among different demographic groups, including differences in gender, age, education level, household registration, health status, and income status. Overall, this study provides strong evidence for the health-related economic costs of air pollution.

## 1 Introduction

Air pollution represents a serious worldwide environmental health threat, with the World Health Organization assessing (49) that 4.2 million deaths annually are caused because of the ambient exposure, primarily through cardiovascular and respiratory diseases^1^. This burden is marked by pronounced socioeconomic disparities: low-income nations experience 23% higher relative healthcare expenditure impacts compared to high-income countries (1), while vulnerable populations face heightened risks due to limited access to protective and mitigation resources (2). The pathophysiological mechanisms underlying these effects are well established. For instance, Pope et al. (3) quantified the dose–response link between PM2.5 exposure and cardiopulmonary mortality, findings supported by Dockery et al. (4) and Samet et al. (5) across diverse populations. Moreover, Brook et al. (6) elucidated biological pathways linking particulate matter (PM) to systemic inflammation and endothelial dysfunction. Recent evidence also highlights developmental consequences: Chay and Greenstone (7) documented increases in infant mortality linked to recession-induced pollution shocks, while Currie et al. (8) identified long-term health and economic impacts associated with early-life exposure.

Beyond conventional health outcomes, air pollution significantly shapes behavioral responses and economic decision-making. Neidell (9) pioneered research on avoidance behaviors, demonstrating reductions in asthma hospitalizations during ozone alerts, while Zivin and Neidell (10) quantified productivity losses attributable to pollution exposure. Defensive strategies also play an important role, with Ito and Zhang (11) measuring Chinese households’ “willingness-to-pay for cleaner air,” and Deschenes et al. (12) analyzing defensive expenditures under the NOx Budget Program. These individual responses further accumulate into broader macroeconomic effects. For example, Hanna and Oliva (13) estimated a 5.8% reduction in labor supply during peak pollution episodes in Mexico City, Greenstone and Hanna (14) reported declines in infant mortality following Indian environmental regulations, and Walker (15) quantified sectoral reallocation costs resulting from compliance with the U. S. Clean Air Act. Liao et al. (16) found that environmental pollution significantly exacerbates income related health inequalities, thereby further widening the wealth gap. Furthermore, Liao et al. (17) found that controlling air pollution can significantly improve residents’ physical health.

The psychological and societal ramifications of air pollution reveal complex welfare trade-offs. Braithwaite et al. (18) identified PM2.5 thresholds above 50 μg/m^3^ as increasing depression risk by 10%, potentially influencing migration dynamics, as Chen et al. (19) observed a 2.8% population outflow for every 10% rise in PM2.5 concentrations. Welfare valuation research further underscores these impacts: Welsch (20) equated PM10 reductions to income-equivalent gains of 11% in life satisfaction, whereas Neidell (21) reported that pollution alerts paradoxically increased private vehicle use. More recent studies have broadened the lens to quality-of-life dimensions. Wei et al. (22) found that long-term exposure to environmental air pollution is associated with an increased incidence of depression among the Chinese population, further revealing the profound impact of air pollution on mental health. Ren et al. (23), on the other hand, from the perspectives of residents’ attention and the sustainability of commercial health insurance, how does air pollution affect the complex trade-off between social mental health and economic behavior. Zhang et al. (24) demonstrated a strong negative link between subjective well-being and PM2.5 exposure in China, with each 1 μg/m^3^ increase linked with a 0.8 percentage point decline in life satisfaction. Fertility behavior has also been shown to respond to environmental conditions; Zhang and Yu (25) found that air pollution lowered fertility intentions by 12.7% among women of childbearing age in highly polluted areas. Additionally, emerging evidence suggests physiological pathways, with Zhang et al. (26) indicating that metabolic disruptions caused by PM may contribute to obesity. Urbanization further complicates these dynamics: Schlenker and Walker (27) linked airport-related emissions to localized health declines, while Banzhaf and Walsh (28) provided empirical support for Tiebout’s “voting with feet” migration hypothesis.

Physical activity represents a critical behavioral nexus where the health benefits of exercise intersect with the risks posed by pollution exposure. Lin et al. (29) research indicates that in China, the cardiovascular health benefits of active commuting are moderated by environmental fine particulate matter, highlighting the complex health trade-off between physical activity and pollution exposure. The study by Hao et al. (30) analyzed the PURE-China cohort and pointed out the combined impact of long-term exposure to outdoor fine particulate matter and physical activity on mortality and cardiovascular events, further confirming physical activity as a key behavioral node where health benefits and pollution risks converge (29, 30). Rundell (31) demonstrated that PM2.5 deposition during exercise is 5–20 times higher than at rest. At the population level, behavioral adaptations are evident: Roberts et al. (32) documented an inverse link between physical activity and pollution, while Yu et al. (33) quantified that retirees reduced weekly walking by 4.69 h for every 56.6 μg/m^3^ raise in PM2.5. These behavioral responses present a fundamental challenge to policy initiatives such as China’s “Healthy China Strategy,” promoted at the 19th CPC National Congress, which emphasizes increased physical activity as a cornerstone of public health.

Policy interventions have demonstrated measurable yet uneven success in addressing air pollution. Shapiro and Walker (34) attributed 60% of U. S. manufacturing emissions reductions to regulatory measures, while Greenstone (35) documented technology-driven substitutions prompted by the Clean Air Act. Similarly, Davis (36) quantified the influence of power plant proximity on property values. Nonetheless, persistent challenges remain in developing countries. Ebenstein et al. (37) revealed stark mortality disparities caused by China’s Huai River policy, Barwick et al. (38) measured morbidity costs through consumer spending patterns, and Luechinger (39) identified significant life satisfaction effects from transboundary pollution. Innovative monitoring and analytical approaches continue to expand the policy toolkit. For instance, Apte et al. (40) leveraged Google Street View mapping to refine urban pollution monitoring, while Deryugina et al. (41) used wind-direction variation as a usual experiment to improve causal identification of pollution’s health impacts. Bruyneel et al. (42) evaluated the positive impacts of the low-emission zone policies in Antwerp and Brussels on air quality, socio-economic disparities and health conditions through quasi-experimental methods. New empirical evidence was provided for the effectiveness of policy intervention. Liu et al. (43) evaluated the effect of China’s air pollution control policies using the different-in-differences method, revealing the significant but uneven effectiveness of policy intervention in improving air quality. Ren et al. (44) found that innovative urban policies can provide strong and effective support for air pollution control.

There is a lack of empirical research in academia on the impact of air pollution on residents’ fitness behavior. Our study will serve as a supplement to this area of research. This study advances the understanding of pollution–behavior interactions through three key innovations. First, we employ temperature (temp) inversion as a natural experiment (45) to address endogeneity concerns that have limited prior observational studies (46). Second, we integrate high-resolution pollution data from NASA’s MERRA-2 database (47) with longitudinal China family panel studies (CFPS) fitness behavior records (48) which represents an innovative approach to bridging macro-level environmental monitoring with micro-level behavioral data. By combining satellite-derived, grid-level PM2.5 concentration estimates with detailed individual exercise habits tracked over multiple years, this integration enables a granular analysis of how localized air pollution variations directly influence personal fitness decisions— a methodological advancement that transcends traditional reliance on coarse administrative pollution data or isolated datasets. Third, our findings inform the implementation of the “Healthy China” strategy by identifying context-specific barriers to physical activity in polluted urban regions, thereby complementing global evidence on avoidance behaviors and providing policy-relevant elasticity estimates for Chinese urban planners. Ultimately, this study is expected to provide experiential insights for environmental governance departments, social security departments, and fitness related institutions.

## 2 Model specification

This study sought to examine the causal link between residents’ fitness behavior and air pollution. In the baseline analysis, PM2.5 annual average concentrations (AACs) are used as a proxy variable for atmospheric pollution, while SO_2−_ ACCs are introduced as an alternative proxy in robustness checks. The empirical results may be affected by endogeneity issues arising from three main sources. First, omitted variable bias may distort the findings. For example, economic development is typically positively correlated with air pollution, as more developed regions often experience higher pollution levels. At the same time, economic development shapes residents’ behavioral patterns, influencing lifestyle choices and fitness participation. Consequently, individuals may relocate based on personal preferences, economic opportunities, and social characteristics, thereby systematically altering their exposure to pollution. Second, reverse causality between air pollution and fitness behavior may bias results. Residents’ physical fitness influences social productivity, and higher productivity may increase pollutant emissions, which in turn worsen air quality. Third, measurement error in pollution indicators may occur, as air quality data in some countries are vulnerable to deliberate manipulation. To address this, the study relies on data from reputable and verified sources to minimize such risks.

Temperature inversion, a well-documented meteorological phenomenon, exhibits a strong correlation with pollution levels and is therefore used as an instrumental variable (IV) to address endogeneity. The temp inversion measure satisfies the exogeneity conditions required for a valid instrument, and its use in air pollution research is well established in both domestic and international literature (45). To further strengthen identification, we incorporate a comprehensive set of meteorological covariates, including daily mean temp, sunshine duration, maximum (max) and minimum (min) temps, wind speed and direction, and precipitation. Controlling for these factors ensures that the temp inversion variable satisfies the exclusion restriction required for instrumental variables (IVs). Accordingly, we specify the following two-stage least squares (2SLS) econometric model:


(1) $${\text{Exercise}}_{\mathrm{ict}}=\alpha_{0}+\alpha_{1}{\mathrm{Air}}_{\mathrm{ict}}+\beta X_{\mathrm{ict}}^{'}+\gamma W_{\mathrm{ict}}^{'}+\lambda_{i}+\mu_{t}+\varepsilon_{\mathrm{ict}}$$

(2) $${\mathrm{Air}}_{\mathrm{ict}}=\varphi_{0}+\varphi_{1}{\mathrm{TI}}_{\mathrm{ict}}+\beta X_{\mathrm{ict}}^{'}+\gamma W_{\mathrm{ict}}^{'}+\lambda_{i}+\mu_{t}+\varepsilon_{\mathrm{ict}}^{'}$$

${\text{Exercise}}_{\mathrm{ict}}$ represents the weekly frequency of fitness activities for individual i, where c denotes the county and t the year. This measure captures respondents’ fitness behavior across time, geographic regions, and age groups. The variable ${\mathrm{Air}}_{\mathrm{ict}}$ reflects ambient air quality in county c during year t, with PM2.5 concentration serving as the primary regressor in the baseline specification.

The variable $X_{\mathrm{ict}}^{'}$ represents the influence of individual and socioeconomic characteristics on ${\text{Exercise}}_{\mathrm{ict}}$ , including age, educational attainment, marital status, self-rated health, residential location, employment status, health insurance coverage, income level, and overall socioeconomic status. The term ${\mathrm{\gamma W}}_{\mathrm{ict}}^{'}\;$ captures the effects of weather-related factors, such as annual average temp, daily min and max temps, sunshine duration, wind speed and direction, as well as precipitation levels. The IV ${\mathrm{TI}}_{\mathrm{ict}}$ denotes the annual frequency of temp inversion events in county c during year t. To address unobserved heterogeneity, $\lambda_{i}\;$ represents individual fixed effects, accounting for time-invariant features like gender, while $\mu_{t}\;$ denotes time fixed effects, capturing common temporal shocks (e.g., the “Healthy China Strategy” proposed during the “19th National Congress of the Communist Party of China,” which significantly increased public awareness of physical fitness and exercise). Finally, $\varepsilon_{\mathrm{ict}}$ represents the idiosyncratic error term.

## 3 Empirical methodology

### 3.1 Data specification

This study draws on data related to urban air pollution, atmospheric temperature inversion indicators, and the China Family Panel Studies (CFPS), a national survey conducted by the Institute of Social Science (ISS) at Peking University from 2010 to 2018. The CFPS, supported by Peking University, was carried out in 2010, 2012, 2014, 2016, and 2018. It covers a broad range of research topics, including physical exercise, employment, education, health, economic activities, social participation, family dynamics, and grassroots governance. The CFPS is characterized by comprehensive coverage, methodological rigor, and a large sample size, ensuring strong representativeness. Specifically, the baseline surveys for 2010–2018 were drawn from 23 provinces using a three-stage sampling procedure (county, village, and household), ultimately encompassing tens of thousands of respondents.

This paper relies on CFPS data for three key reasons. First, the baseline survey consistently included questions about respondents’ physical exercise habits, specifically the number of weekly exercise sessions. Importantly, this question was repeated across all five survey waves (2010–2018), making exercise frequency a reliable dependent variable for this study. Second, the CFPS dataset contains precise information on residential location and interview dates, enabling accurate spatiotemporal matching between respondents’ exercise data and local annual air pollution levels. Third, the CFPS provides extensive data on multidimensional individual features, including education’s years, age, residential location, employment status, marital status, and self-assessed health status, enabling the inclusion of a robust set of control variables in the econometric analysis.

This study employs county-level PM2.5 AACs to represent regional air pollution levels. Currently, the main source of air pollution data in China is provided by ground-based monitoring stations, which are maintained by the China National Environmental Monitoring Centre (CNEMC), an agency under the Ministry of Ecology and Environment. The key advantage of this dataset is its relatively high measurement accuracy; however, it also presents several drawbacks, including the limited number of stations, their uneven geographic distribution, and the high costs associated with monitoring. Moreover, monitoring station data cannot fully capture individual pollutants, and PM2.5 concentration values may be subject to potential manipulation. To mitigate potential bias arising from these data limitations, this study employs satellite-derived aerosol optical depth (AOD) data to estimate near-surface PM2.5 concentrations. The underlying principle is that AOD represents the vertical integral of the extinction coefficient throughout the atmospheric column and exhibits a strong correlation with ground-level PM concentrations. Following the methodology of Buchard et al. (47), we obtained longitude–latitude gridded AOD data (0.5° × 0.625°, around 60 km × 50 km) dating back to 1980 from version 5.12.4 of the M2TMNXAER product, publicly released by the NASA. In line with Buchard et al. (47), ArcGIS 10.2 software was then used to calculate gridded PM2.5 ACC data across all of China for the period 2010–2018. These annual pollutant data were subsequently aggregated at the county level. In addition, NASA provides SO_2−_ ACC data, which allows this study to construct county-level annual average SO_2−_ values using the same methodology, thereby serving as a proxy variable for air pollution in robustness checks.

Additionally, this study incorporates several meteorological indicators as control variables. Moreover, the weather data, obtained from 820 meteorological stations across China and provided by the “China Meteorological Administration Information Center,” include daily average temp, max and min temps, sunshine duration, wind direction and speed, and precipitation. To construct county-level annual averages, the inverse distance weighting (IDW) method was applied to interpolate meteorological information from station points, using a search radius of 200 kilometers.

This study further employs the annual frequency of temp inversion events as an IV for air pollution. The inversion data were sourced from the second Modern-Era Retrospective analysis for Research and Applications, Version 2 (MERRA-2) dataset released by NASA. MERRA-2 provides atmospheric temp profiles from 110 meters to 36,000 meters above ground level since 1980, reported as six-hourly averages across 42 atmospheric pressure layers within 50 km × 60 km grid cells. For each year and each pressure layer, the six-hourly values were aggregated from the grid level to the county level. Under typical conditions, surface temps are higher than those in upper layers, indicating the absence of inversion. In contrast, a temp inversion is identified when the temp in the lowest atmospheric layer (110 meters above ground level) is higher than that in the second layer (320 meters above ground level). During such events, air pollutants remain trapped near the surface and fail to disperse efficiently, thereby exacerbating pollution levels. Given that MERRA-2 provides six-hourly averages, inversion conditions can be identified up to four times daily. To ensure consistency and reduce measurement error, this study defines a daily inversion occurrence if at least one inversion event is detected within a 24-h period. The total annual count of inversion days was then calculated for each county.

### 3.2 Variable statistical description

As shown in Table 1, the variable Exercise reflects respondents’ weekly physical exercise behavior (0 = no exercise, 1 = exercises). Health measures self-rated health status, with values from 1 to 5, where higher scores specify greater satisfaction with one’s current physical health. Urban Hukou denotes whether the respondent holds an urban household registration (1 = urban hukou, 0 = rural hukou). Age represents the respondent’s age, while Years of Education captures educational attainment. Marital Status records the respondent’s marital situation (Widowed = 5, Divorced = 4, Cohabiting = 3, Married = 2, and Single = 1). Employment Status indicates work participation (0 = unemployed, 1 = employed), and Insurance shows whether the respondent has insurance coverage (0 = no insurance, 1 = insured). Income satisfaction captures individuals’ satisfaction with their income levels, measured on a scale from Very satisfied (5) to Very dissatisfied (1). Social standing reflects respondents’ self-perceived social status within their local community, ranging from Very high (5) to Very low (1). Environmental variables include PM2.5, representing the AAC of fine PM (μg/m^3^), and SO_2−_, representing the AAC of sulfur dioxide (μg/m^3^). Inversion Days 1 measures the annual count of days with temperature inversions occurring between the second atmospheric layer (320 meters) and the first layer (110 m), whereas Inversion Days 2 quantifies inversions between the first layer (110 m) and the third layer (540 m). Within the study sample, 61.2% of respondents reported engaging in weekly exercise. From 2010 to 2018, the average annual PM2.5 concentration was 72.44 μg/m^3^, surpassing the WHO health safety guideline of 10 μg/m^3^ by more than sevenfold. The standard deviation (SD) of PM2.5 concentration was 31.18 μg/m^3^, with observed values ranging from 7.292 μg/m^3^ to 141.6 μg/m^3^. Regarding atmospheric inversions, the mean annual count of inversion days between the first and second layers was 158.1, indicating inversions occurred on nearly half the days of the year, with a SD of 77.69 days, a min of 4 days, and a max of 324 days. For inversions between the first and third layers, the average annual count was 112.8 days, with a SD of 60.69 days, a min of 1 day, and a max of 275 days.

**Table 1:** Descriptive statistics.

Variable	Average value	SD	Min value	Max value
Keep-fit exercises	0.612	0.487	0	1
Health	2.933	1.305	1	5
Whether in the town	0.443	0.497	0	1
Age	50.45	13.67	16	94
Years of education	6.435	4.741	0	20
Marriage	2.148	0.745	1	5
Employment	0.678	0.467	0	1
Insurance	0.914	0.280	0	1
Income	2.491	1.045	1	5
Social class	2.931	1.051	1	5
PM2.5	72.44	31.18	7.292	141.6
SO_2_	26.20	15.12	0.816	63.60
Temp	14.31	4.701	−0.900	23.70
Humidity	66.00	9.108	42.40	86
Precipitation	946.9	543.0	132.2	3,203
Sunshine duration	1971	477.0	598.4	3,024
Days with adverse weather 1	158.1	77.69	4	324
Days with adverse weather 2	112.8	60.69	1	275

## 4 Results of empirical evidence

### 4.1 OLS regression outcomes

Table 2 presents the results that was carried out exclusively by employing the Ordinary Least Squares (OLS) regression model defined in Equation 1. Column (1) reports regression findings excluding both individual-level and weather control variables. Column (2) excludes individual-level controls but incorporates weather controls. Besides, column (3) contains individual-level controls but omits weather controls, while column (4) comprises both individual-level and weather control variables.

**Table 2:** OLS regression results without IVs.

Dependent variable: fitness exercise	(1)	(2)	(3)	(4)
PM2.5 (μg/m3)	0.0000 (0.0006)	0.0011 (0.0007)	−0.0001 (0.0006)	0.0006 (0.0008)
Age			−0.0123* (0.0068)	−0.0036 (0.0115)
Education			−0.0015 (0.0016)	−0.0018 (0.0017)
Whether in the town			−0.0021 (0.0126)	−0.0063 (0.0142)
Marriage			0.0062 (0.0061)	0.0047 (0.0068)
Employment			0.0364 (0.0056)	0.0319 (0.0063)
Health			0.0055	0.0071
Insurance			(0.0022) 0.0002	(0.0026) −0.0082
Income			(0.0078) −0.0005	(0.0090) −0.0027
Social class			(0.0024) −0.0109 (0.0023)	(0.0028) −0.0101 (0.0028)
Meteorological control	×	√	×	√
Individual FE	√	√	√	√
Year FE	√	√	√	√

Heteroskedasticity-robust standard errors are presented in parentheses and * denotes significance at the 10% significance level.

The results demonstrate that, across all model specifications, no statistically significant negative association between air pollution and individuals’ engagement in physical exercise, irrespective of whether weather-related or individual-level controls are included. This outcome suggests that potential endogeneity may be introducing bias into the estimates. To address this concern, the research uses the annual count of temp inversion days as an IV for air pollution and applies a 2SLS estimation strategy.

### 4.2 2SLS regression outcomes

#### 4.2.1 First-stage regression results: the influence of temp inversion days on air pollution

The objective of the first-stage test is to examine whether temp inversions influence air pollution, with the results reflecting the extent to which the frequency of inversions affects pollution levels. As shown in Equation 2, the frequency of temp inversions is included as the independent variable, while PM2.5 concentration serves as the dependent variable. The *F*-statistics are all greater than 10, which confirms the overall statistical significance of the model. As shown in Table 3, column (1) reports the regression outcomes excluding both individual-level and meteorological control variables. Column (2) includes meteorological controls but excludes individual-level controls. Column (3) incorporates individual-level controls while excluding meteorological controls, and column (4) presents the regression outcomes including both sets of controls.

**Table 3:** Regression outcomes for Phase One.

Dependent variable: PM (2.5 μg/m^3^)	(1)	(2)	(3)	(4)
Temp inversion (annual Occurrence frequency)	0.0107*** (0.0008)	0.0071*** (0.0009)	0.0102*** (0.0008)	0.0066*** (0.0009)
Individual-level control variables	×	×	√	√
Meteorological control	×	√	×	√
Individual FE	×	√	√	√
Year FE	×	√	√	√
F-statistics	185.87	1981.75	20.92	668.32

Heteroskedasticity-robust standard errors are presented in parentheses and *** denotes significance at the 1% significance level.

The first row of Table 3 shows a statistically significant positive link between the frequency of temp inversions and PM2.5 AACs across all specifications. In particular, a higher number of inversion days within a year is associated with elevated annual average PM2.5 levels. This finding supports the theoretical expectation that, during inversion episodes, atmospheric pollutants are trapped near the surface and fail to disperse effectively, thereby worsening air quality in affected regions. The estimated coefficient of temp inversion, 0.0066, indicates that each additional inversion day surges the PM2.5 AAC by 0.0066 μg/m^3^.

#### 4.2.2 Second-stage regression results: the impact of air pollution on residents’ physical activity behaviors

As shown in Table 4, the PM2.5 AAC is employed as the key explanatory variable to measure air pollution levels, and the second-stage regression outcomes are reported. Column (1) shows the regression estimates without individual-level or weather-related variables, but with both individual and year fixed effects included. The results reveal a statistically significant negative association at the 1% significance level between PM2.5 AACs and residents’ physical exercise behavior, with a regression coefficient of −0.0391. Column (2) extends this specification by adding weather controls, and while the negative relationship remains statistically significant (1% level), the coefficient magnitude increases in absolute terms to −0.0614. Column (3), which incorporates individual-level controls based on column (1), continues to show a significant negative effect (1% level), with a coefficient of −0.0397, nearly identical to the value reported in Column (1). Column (4), which comprises both individual-level and weather -related control variables, also reports a statistically significant negative association at the 1% significance level, with a coefficient of −0.0660. These results highlight that individual-level control variables exert little influence on the PM2.5 estimated effect, whereas the inclusion of weather variables has a more substantial impact on the coefficient magnitude. Specifically, the column (4) estimates propose that a 1 μg/m^3^ rise in PM2.5 AACs is associated with a 6.6% reduction in the probability of residents engaging in weekly physical exercise. Considering the SDs of 0.487 for the dependent variable (physical exercise) and 31.18 for the explanatory variable (PM2.5), the standardized regression coefficient is calculated as −4.23, indicating that in column (4), a one- SD rise in annual average PM2.5 level is associated with a 4.23-SD decline in weekly physical exercise.

**Table 4:** Regression outcomes of the Second Stage.

Dependent variable: fitness exercise	(1)	(2)	(3)	(4)
PM2.5 (μg/m3)	−0.0391*** (0.0077)	−0.0614*** (0.0163)	−0.0397*** (0.0082)	−0.0660*** (0.0181)
Age			−0.0129* (0.0067)	−0.0058 (0.0106)
Education			−0.0015 (0.0016)	−0.0016 (0.0019)
Whether in the town			0.0216 (0.0144) 0.0063	0.0382* (0.0205) 0.0044
Marriage			(0.0065) 0.0303***	(0.0077) 0.0285***
Employment			(0.0060) 0.0035	(0.0070) 0.0053*
Health			(0.0023) 0.0068	(0.0029) −0.0013
Insurance			(0.0083) −0.0011	(0.0106) −0.0051
Income			(0.0025) −0.0120***	(0.0032) −0.0107***
Social class			(0.0024)	(0.0031)
Meteorological control	×	√	×	√
Individual FE	√	√	√	√
Year FE	√	√	√	√

Heteroskedasticity-robust standard errors are presented in parentheses. Statistical significance is indicated by * and *** at the 10% and 1% levels, respectively.

Furthermore, the regression outcomes in column (4) show a statistically significant positive association at the 10% level between urban household registration status and residents’ exercise behavior. This result indicates that urban residents tend to participate more in physical fitness activities, which may be attributed to the higher availability of exercise facilities and a broader range of recreational opportunities in urban areas compared to rural regions. Such factors may encourage stronger interest and participation in physical activity. In addition, the ongoing process of urbanization has facilitated the transition of rural residents into urban populations, thereby broadening the base of individuals who regularly engage in exercise. The analysis also reveals a statistically significant positive correlation at the 1% significance level between employment status and exercise participation, indicating that higher employment rates contribute to greater involvement in fitness activities. Moreover, health status demonstrates a positive relationship with exercise behavior at the 10% significance level, suggesting that individuals who report higher satisfaction with their health are more likely to participate in physical activity. Finally, the results identify a statistically significant negative association at the 1% level between social status and residents’ exercise behavior.

## 5 Robustness test

Drawing on the above findings, air pollution exhibits a negative association with residents’ physical activity behavior. To verify the validity of this conclusion, this section conducts a series of robustness checks.

In the 2SLS estimation, the AAC of PM2.5 was employed as the primary explanatory variable representing air pollution levels. To further strengthen the results, this section adopts the AAC of SO_2−_ as an alternative indicator of air pollution severity.

As demonstrated in Table 5A, the first-stage regression outcomes demonstrate that, across all model specifications, temp inversion has a statistically significant positive association with SO_2−_ AACs at the 1% level, suggesting that a higher frequency of annual temp inversion events corresponds to increased SO_2−_ pollution levels, which is consistent with established atmospheric science principles.

**Table 5:** First- and second-stage regression results for air pollution (SO2) and fitness behavior.

Panel A				
Dependent variable: SO_2−_ (μg/m3)	(1)	(2)	(3)	(4)
Temp inversion frequency (annual occurrences)	0.0314*** (0.0004)	0.0400*** (0.0004)	0.0028*** (0.0004)	0.0036*** (0.0004)
Individual-level controls	×	×	√	√
Meteorological controls	×	√	×	√
Individual FE	√	√	√	√
Year FE	√	√	√	√
*F*-statistic	78.7	536.91	12.19	174.33

Panel B				
Dependent variable: physical exercise participation	(1)	(2)	(3)	(4)
SO_2−_ (μg/m3)	−0.1353*** (0.0294)	−0.1110*** (0.0285)	−0.1439*** (0.0333)	−0.1207*** (0.0320)
Individual-level controls	×	×	√	√
Meteorological controls	×	√	×	√
Individual FE	√	√	√	√
Year FE	√	√	√	√

Heteroskedasticity-robust standard errors are presented in parentheses and *** denotes significance at the 1% significance level.

This outcome further supports the proposed mechanism: temp inversions trap pollutants near the surface and hinder their vertical dispersion, thereby intensifying air pollution severity in affected regions.

All F-statistics are above 10, with the F-statistic from Specification (4) reaching 174.33, indicating strong instrument relevance at conventional significance levels. The estimated coefficient for temp inversion frequency is 0.0036 (*p* < 0.01), indicating that each additional inversion day surges SO_2−_ AACs by 0.0036 μg/m^3^, an effect that carries both statistical significance and practical implications for environmental policy.

Table 5B reports the second-stage regression outcomes. Column (4) shows that an increase of 1 μg/m^3^ in SO_2−_ ACCs is associated with a 12.07% decrease in the percentage of residents who participate in weekly physical exercise. Referring to Table 1, the SDs of the dependent variable (exercise participation) and the independent variable (SO_2−_) are 0.487 and 15.12, respectively. Given a regression coefficient of −0.1207, the corresponding standardized beta coefficient is calculated as −3.82. This result implies that a one-SD rise in SO_2−_ concentration is linked with a 3.82-SD decline in weekly exercise participation.

Second, the robustness of the IV is conducted by redefining inversion frequency using alternative atmospheric layers. While the baseline specification measured inversions between the first layer (110 m) and the second layer (320 m), this section employs inversions occurring between the first layer (110 m) and the third layer (540 m) as the IV in the 2SLS estimation.

The results presented in Table 6 demonstrate:

(1) The first-stage regression outcomes confirm a statistically significant positive association between thermal inversions and air pollution.
(2) The second-stage regression results demonstrate a statistically significant negative association between air pollution levels and the physical exercise behavior of residents.

**Table 6:** First- and second-stage regression results for alternative inversion layer definition.

Panel A				
Dependent variable: PM2.5_−_ (μg/m3)	(1)	(2)	(3)	(4)
Thermal Inversions (annual count, 110–540 m)	0.0113*** (0.0008)	0.0040*** (0.0008)	0.0104*** (0.0008)	0.0030*** (0.0009)
Individual-level controls	×	×	√	√
Meteorological controls	×	√	×	√
Individual FE	√	√	√	√
Year FE	√	√	√	√
*F*-statistic	185.87	1981.75	20.92	668.32

Panel B				
Dependent variable: physical exercise participation	(1)	(2)	(3)	(4)
PM2.5 (μg/m3)	−0.0817*** (0.0100)	−0.2628*** (0.0663)	−0.0904*** (0.0113)	−0.3499*** (0.1098)
Individual-level controls	×	×	√	√
Meteorological controls	×	√	×	√
Individual FE	√	√	√	√
Year FE	√	√	√	√

Heteroskedasticity-robust standard errors are presented in parentheses and *** denotes significance at the 1% significance level.

These findings remain consistent with our baseline regression conclusions, though coefficient magnitudes differ. The robustness of our IV approach is therefore empirically validated.

Finally, a falsification test is conducted by estimating a 2SLS model using respondents’ height as the dependent variable. Height is chosen as a placebo outcome because it is theoretically exogenous to air pollution exposure; unlike physical exercise behavior, no statistically significant relationship is expected.

The regression outcomes revealed in Table 7 confirm that air pollution does not have a statistically significant impact on height. This finding provides additional support for the validity of our main conclusion, indicating that the observed negative association between air pollution and residents’ physical exercise behavior likely reflects a causal effect rather than a false association.

**Table 7:** Falsification test (placebo regression).

Dependent variable: height	(1)	(2)	(3)	(4)
2SLS estimates: PM2.5(μg/m3)	0.0383 (0.0805)	−0.0643 (0.1363)	−0.0264 (0.0890)	−0.1647 (0.1555)
Individual-level controls	×	×	√	√
Meteorological controls	×	√	×	√
Individual FE	√	√	√	√
Year FE	√	√	√	√

Heteroskedasticity-robust standard errors are presented in parentheses.

## 6 Heterogeneity analysis

This section investigates the heterogeneous impacts of air pollution on residents’ physical exercise behavior across personal characteristics, including gender, age, educational attainment, and household registration.

Table 8 highlights notable heterogeneity in the impact of air pollution across demographic groups. Columns (1) and (2) show that air pollution exerts a more pronounced negative effect on women’s exercise behavior compared with men, as reflected by a larger correlation coefficient for women. This difference may stem from variations in physical constitution and sensitivity to environmental factors, as women may be more attentive to air pollution concerns and physiologically more affected by its adverse health impacts.

**Table 8:** Analysis results of gender and age heterogeneity.

The regression outcomes of the second stage	Gender		Age		
Explained variable: fitness exercise	Female	Male	Young people (<39)	Wrinkly (40–59)	Old people (≥60)
	(1)	(2)	(3)	(4)	(5)
PM2.5(μg/m3)	−0.1304*** (0.0465)	−0.0311* (0.0175)	−0.0553** (0.0268)	−0.0520*** (0.0197)	−0.1238 (0.1313)
Individual-level control	√	√	√	√	√
Weather control	√	√	√	√	√
Individual FE	√	√	√	√	√
Year FE	√	√	√	√	√
*F*-statistic	346.69	325.77	155.35	391.43	133.4

Heteroskedasticity-robust standard errors are presented in parentheses. Statistical significance is indicated by *, **, and *** at the 10, 5, and 1% levels, respectively.

The findings in Columns (3)–(5) indicate that air pollution significantly affects exercise behavior at the 5% level for younger adults, at the 1% level for middle-aged adults, and is not statistically significant for older adults. These differences may be attributed to three main factors. First, younger and middle-aged adults typically spend more time outdoors than older adults, resulting in greater exposure to air pollution. Second, they have better access to pollution-related information, enabling timely awareness of air quality updates through the internet. Third, older adults, having lived in the area for extended periods, may develop stronger physiological adaptation to local environmental conditions, thereby reducing their perceived sensitivity to pollution.

Table 9 presents the heterogeneous effects of education level and hukou status on exercise behavior. Columns (1)–(2) show that, when respondents are grouped by education level, the coefficient for the higher-educated group is significant (1% level), while that for the lower-educated group is significant (5% level). This implies that higher educational attainment intensifies the adverse effect of air pollution on residents’ physical fitness behavior, possibly because more educated individuals are more aware of the health risks related with pollution. Columns (3)–(4) categorize respondents by hukou status, revealing further disparities. The results show that air pollution has a statistically significant impact on the exercise behavior of rural residents at the 10% significance level, whereas no significant effect is observed for urban residents. This difference may be due to rural residents’ limited access to indoor exercise facilities, requiring them to engage in outdoor activities where exposure to pollution is unavoidable, while urban residents can more easily shift to indoor exercise settings.

**Table 9:** Heterogeneous effects by education level and Hukou status.

Second-stage regression outcomes	Years of schooling		Whether in the town	
Dependent variable: fitness exercise	<9 years	≥9 years	No	Yes
	(1)	(2)	(3)	(4)
PM2.5(μg/m3)	−0.0639** (0.0323)	−0.0666*** (0.0222)	−0.0637* (0.0379)	−0.0156 (0.0113)
Individual-level control	√	√	√	√
Weather control	√	√	√	√
Individual FE	√	√	√	√
Year FE	√	√	√	√
*F*-statistic	344.74	309.9	287.37	488.19

Heteroskedasticity-robust standard errors are presented in parentheses. Statistical significance is indicated by *, **, and *** at the 10, 5, and 1% levels, respectively.

Table 10 presents the results of a heterogeneity analysis on residents’ health status and income. The results in columns (1) and (2) indicate that air pollution has a stronger and highly significant negative effect on the fitness behavior of individuals with better health (at the 1% significance level), whereas its impact on individuals with poorer health is minimal. This difference can be explained by the fact that individuals with better health generally have a more active intention and higher frequency of engaging in fitness activities. Consequently, they are more sensitive to the negative effects of air pollution on outdoor fitness. In contrast, individuals with poorer health are less likely to participate in regular fitness activities, making them less affected by air pollution. The results in columns (3) and (4) reveal that the fitness behavior of low-income individuals is significantly more affected by air pollution (at the 1% significance level), while high-income individuals are largely unaffected. A possible explanation is that low-income individuals tend to engage in fitness activities in free, open-air environments, which are more susceptible to air pollution. In contrast, high-income individuals are more likely to use paid, professional indoor fitness facilities, which provide a controlled environment and thus reduce their exposure to polluted air.

**Table 10:** Heterogeneity analysis results of health and income.

Second-stage regression outcomes	Health		Income	
Dependent variable: fitness exercise	Poor	Good	Low	High
	(1)	(2)	(3)	(4)
PM2.5(μg/m3)	0.0006 (0.0277)	−0.0991*** (0.0227)	−0.0465*** (0.0164)	−0.0259 (0.0531)
Individual-level control	√	√	√	√
Weather control	√	√	√	√
Individual FE	√	√	√	√
Year FE	√	√	√	√
*F*-statistic	21.13	44.59	59.34	6.03

Heteroskedasticity-robust standard errors are presented in parentheses and *** denotes significance at the 1% significance level.

## 7 Conclusion and policy recommendations

This study utilized data from the China Family Panel Studies (CFPS) from 2010 to 2018, combined with local air quality and meteorological records of the corresponding years, to accurately measure the frequency of physical exercise among respondents. To address the potential endogeneity of air pollution exposure, this study employed the meteorological phenomenon of temperature inversion as an instrumental variable. Through two-stage least squares (2SLS), this study empirically established the causal relationship between air pollution and residents’ physical exercise behavior.

The research results show that for every one standard deviation increase in the annual average concentration of PM2.5, the frequency of residents’ physical exercise decreases by 4.23 standard deviations. To test the robustness, this study first used the annual average concentration of sulfur dioxide as an alternative estimate of air pollution. Additionally, by modifying the selection criteria for temperature inversion and conducting placebo tests using respondents’ height instead of physical exercise behavior, the analysis results were further verified. Heterogeneity analysis indicates that the adverse impact of air pollution on physical exercise behavior is more significant among women, young and middle-aged people, those with higher education levels, non-urban residents, people in good health, and low-income groups. The above analysis suggests that air pollution has now become a key factor affecting residents’ willingness to exercise. Therefore, in promoting the national fitness program and the Healthy China initiative, it is necessary to address the ecological and environmental challenges related to air pollution. Based on these findings, the following suggestions are proposed:

First, in the process of advancing ecological civilization construction and sustainable development, it is recommended to strengthen the air quality monitoring system and establish a risk warning mechanism to overcome the public’s cognitive barriers to pollution exposure. In international comparisons, many developed countries, such as the United States and the United Kingdom, have well-established air quality monitoring and warning systems. The United States Environmental Protection Agency (USEPA) has established a national air quality monitoring network that can provide real-time and accurate data on the concentrations of relevant air pollutants, which helps the public obtain air quality information in a timely manner. Therefore, the government should intensify efforts to improve air quality, improve the monitoring mechanism, ensure the reliability and accuracy of data, and promptly release air quality information to the public, enabling residents to take appropriate protective measures.

Second, it is necessary to enhance public awareness of the concept that “green mountains and clear waters are as valuable as mountains of gold and silver,” especially among groups that are more sensitive to air quality. Communities can increase the intensity of health exercise education activities, popularize the hazards of air pollution to health and the protective measures that can be taken among specific groups, and encourage all sectors of society to use new media platforms to disseminate correct fitness awareness and knowledge, explaining the cumulative impact of air pollution on heart and lung function during physical activities in polluted periods.

Third, establish differentiated air pollution control mechanisms to balance health benefits and development needs. Excessively aggressive measures may lead to simplistic solutions, thereby hindering development, while insufficient control may fail to achieve the expected results and improve residents’ living environment. Therefore, it is crucial to establish governance goals based on science. The government can implement corresponding measures based on the pollution levels and industrial characteristics of different regions, establish a flexible seasonal control mechanism based on the threshold of pollution gasses, prioritize restrictions on industrial emissions and traffic pollution during severe pollution periods, and implement dynamic adjustments during non-peak periods. Quantify policy effects through health benefit–cost assessment models to achieve the coordinated optimization of promoting health and economic development.

Fourth, promote the coordinated development of fitness infrastructure and transportation systems to alleviate the impact of spatial limitations caused by environmental factors on health behaviors. Studies have shown that non-urban residents are more vulnerable to pollution, so it is recommended that the government launch special plans to build community fitness centers and prioritize the development of affordable indoor sports facilities in rural areas. In addition, priority should be given to infrastructure development, such as the expansion of high-speed rail networks. By increasing the accessibility of high-speed rail, local pollution can be reduced and travel convenience improved, thereby enhancing residents’ subjective well-being.

Fifth, a coordination mechanism between environmental and health policies should be established to maximize the benefits of pollution control for public health. Improving the ecological environment and controlling air pollution can significantly reduce the incidence of various diseases, including heart disease, lung disease, stroke, and chronic respiratory diseases. These improvements also have a positive impact on the national fitness program, as regular exercise can enhance an individual’s physical resistance and improve their ability to withstand the adverse effects of air pollution. Moreover, promoting fitness activities contributes to economic growth by reducing medical expenses related to pollution-induced diseases and enhancing the overall physical health of the population, thereby boosting social productivity.

## Data Availability

The original contributions presented in the study are included in the article/supplementary material, further inquiries can be directed to the corresponding authors.

## Footnotes

^1^WHO. (2018, May2). Ambient (outdoor) air pollution. Retrieved March14, 2019, from https://www.who.int/en/news-room/fact-sheets/detail/ambient-(outdoor)-air-quality-and-health.
